# Ethnic inequalities in the impact of COVID-19 on primary care consultations: a time series analysis of 460,084 individuals with multimorbidity in South London

**DOI:** 10.1186/s12916-022-02720-7

**Published:** 2023-01-19

**Authors:** Alice McGreevy, Marina Soley-Bori, Mark Ashworth, Yanzhong Wang, Emma Rezel-Potts, Stevo Durbaba, Hiten Dodhia, Julia Fox-Rushby

**Affiliations:** 1 King’s College London, School of Life Course & Population Sciences, Guy’s Campus, Addison House, London, SE1 1UL UK; 2 NIHR Biomedical Research Centre, Guy’s and St Thomas’ NHS Foundation Trust and King’s College London, London, UK; 3 Public Health Directorate, London Borough of Lambeth, London, UK

**Keywords:** Multimorbidity, Primary care, Long-term conditions, COVID-19 pandemic, Ethnic inequalities

## Abstract

**Background:**

The COVID-19 pandemic caused rapid changes in primary care delivery in the UK, with concerns that certain groups of the population may have faced increased barriers to access. This study assesses the impact of the response to the COVID-19 pandemic on primary care consultations for individuals with multimorbidity and identifies ethnic inequalities.

**Methods:**

A longitudinal study based on monthly data from primary care health records of 460,084 patients aged ≥18 years from 41 GP practices in South London, from February 2018 to March 2021. Descriptive analysis and interrupted time series (ITS) models were used to analyse the effect of the pandemic on primary care consultations for people with multimorbidity and to identify if the effect varied by ethnic groups and consultation type.

**Results:**

Individuals with multimorbidity experienced a smaller initial fall in trend at the start of the pandemic. Their primary care consultation rates remained stable (879 (95% CI 869–890) per 1000 patients in February to 882 (870–894) March 2020), compared with a 7% decline among people without multimorbidity (223 consultations (95% CI 221–226) to 208 (205–210)). The gap in consultations between the two groups reduced after July 2020. The effect among individuals with multimorbidity varied by ethnic group. Ethnic minority groups experienced a slightly larger fall at the start of the pandemic. Individuals of Black, Asian, and Other ethnic backgrounds also switched from face-to-face to telephone at a higher rate than other ethnic groups. The largest fall in face-to-face consultations was observed among people from Asian backgrounds (their consultation rates declined from 676 (659–693) in February to 348 (338–359) in April 2020), which may have disproportionately affected their quality of care.

**Conclusions:**

The COVID-19 pandemic significantly affected primary care utilisation in patients with multimorbidity. While there is evidence of a successful needs-based prioritisation of multimorbidity patients within primary care at the start of the pandemic, inequalities among ethnic minority groups were found. Strengthening disease management for these groups may be necessary to control widening inequalities in future health outcomes.

**Supplementary Information:**

The online version contains supplementary material available at 10.1186/s12916-022-02720-7.

## Background

The policy response to the COVID-19 pandemic has greatly impacted the delivery of primary care in the United Kingdom (UK). On 5 March 2020, primary care providers were recommended to change face-to-face consultations to triage appointments via telephone or video to reduce the risk of infection in practices [1]. Older and vulnerable people were advised to shield at home and primary care providers asked to roll out remote consultations to this group as a priority [2]. The first UK national lockdown was announced 23 March 2020, requiring the public to only leave their homes for limited reasons including food shopping and medical needs [3].

Primary care plays a crucial role in caring for individuals with multimorbidity [4, 5]. This group has increased healthcare needs and a higher risk of severe COVID-19 compared to those without multimorbidity [6–9]. The presence of some long-term conditions (LTCs), including hypertension, diabetes and coronary heart disease [10, 11], has been identified as a COVID-19 risk factor, along with sociodemographic characteristics such as being male [12], socially deprived [9], and from ethnic minority groups. Individuals of ethnicity other than White experienced a higher rate of COVID-19 mortality, in particular those from Black or Asian ethnic groups [9, 12, 13]. These risk factors, alongside new barriers to accessing primary care, may have changed healthcare needs and utilisation during the pandemic.

Previous research suggests that ethnicity is an independent contributor to multimorbidity, even after adjusting for social deprivation [14]. The prevalence of multimorbidity varies across ethnic groups and distinct patterns of disease accumulation are observed over time. For example, individuals of Black ethnicity have a higher prevalence of multimorbidity, different patterns of LTC combinations and a more fluctuating disease accumulation pathway compared with those of White ethnicity [15, 16]. Individuals with multimorbidity and particular ethnic groups fared worse during the COVID-19 pandemic, with an increased risk of adverse outcomes due to COVID-19 [9, 12, 13]. However, little is known about how the pandemic affected their use of primary care services. Existing evidence on overall population trends indicates that there was an initial reduction in consultations in April 2020 and face-to-face consultations fell substantially, while telephone and electronic delivery increased [17–19], but whether changes were magnified among those with multimorbidity and from ethnic minority groups remains unexplored.

This paper aims to assess the impact of the response to the COVID-19 pandemic (capturing changes in both policy and patient behaviour) on primary care consultations of individuals with multimorbidity, and identify whether the effects vary by ethnic group. Recognising population subgroups with differential impacts from the pandemic may inform care prioritisation and preparedness for future pandemics to contain the widening of health inequalities.

## Methods

### Study design and population

A longitudinal study design, based on monthly data from primary care health records in the Lambeth DataNet, is used. Lambeth is an inner-city borough in south London which contains an urban, deprived, and multi-ethnic population. The sample includes all patients aged ≥ 18 years who were registered to a general practice in Lambeth (hierarchical data) between the 38-month period from February 2018 to March 2021. The ‘pre-pandemic’ period is defined as February 2018 to February 2020 (25 months) and the ‘pandemic’ period as March 2020 to March 2021 (13 months). Data was not available past March 2021. March 2020 is considered the start of the pandemic reflecting when the UK healthcare system’s response began and the first national lockdown was implemented [1, 3].

### Statistical analysis

Descriptive analyses are conducted for the total monthly consultation rate per 1000 registered patients. This is categorised by provider type and delivery mode to identify changes in the way primary care was provided, and then by multimorbidity status (yes/no).

The effect of the pandemic on primary care consultations is assessed using an interrupted time series (ITS) analysis [20, 21]. The analysis is conducted via a generalised linear model, with a negative binomial distribution and a log-link to account for overdispersion in the number of primary care consultations. Interaction terms to consider both a change in level (the immediate effect) and slope (gradual effect) from the onset of the pandemic in March 2020 are included. The dependent variable is total primary care consultations, with patient-month as the unit of analysis. Total consultations are comprised of three provider types (general practitioner (GP), nurse and other healthcare professionals) and four modes of delivery (face-to-face, telephone, home visits and electronic). Electronic consultations refer to e-mails and other remote online consultations (e.g. e-Consult). Administrative consultations are included in descriptive analyses but excluded from models as they may capture contacts that do not reflect healthcare needs.

The first model specification predicts the effect of the pandemic on total monthly consultations, and analyses whether there are inequalities between those with multimorbidity and those without multimorbidity. Multimorbidity is defined as having two or more of 32 LTCs, selected to reflect demographic and morbidity patterns in an inner-city context [22] (Additional file 1: Table S1). The model (Eq. 1) includes interaction terms between a multimorbidity indicator variable (MM) and the pandemic level and slope change variables to assess differences against those without multimorbidity.

1 $$Consultation{s}_{it}={\beta}_0+{\beta}_1 Time+{\beta}_2 Pandemic+{\beta}_3 Time\ast Pandemic+{\beta}_4{MM}_{it}+{\beta}_5{MM}_{it}\ast Time+{\beta}_6{MM}_{it}\ast Pandemic+{\beta}_7{MM}_{it}\ast Time\ast Pandemic+{\beta}_8{GPpractice}_i+{\beta}_9 Month+{\beta}_{10}{LagRes}_{it}$$

*Time* is the number of months elapsed since the start of the study and captures the linear trend, and *Pandemic* is a dummy variable indicating the pre-COVID-19 period (coded 0) or during the COVID-19 period (coded 1). GP practice includes a set of dummy variables (fixed effects) to account for potential clustering or similarities in consultation rates of patients within the same practice, caused by for example, differences in the size of the workforce and access to technology for remote consultations [23]. Monthly dummy variables were included to adjust for seasonality. *LagRes* is the lagged residuals. Inspection of the autocorrelation and partial autocorrelation functions identified autocorrelation in the dependent variable. To adjust for this, the model was first run without *LagRes*. The residuals from this model were extracted and lagged, and then added as an explanatory variable for the main model. Estimates are given as incidence rate ratios (IRRs).

The second model focusses on the multimorbid population and analyses the variability of the COVID-19 impact by ethnic group. This model has the same specification as Eq. 1, except the multimorbidity indicator is replaced with ethnic group. Classification of ethnicity is based on the 2011 Census and includes seven categories: White, Black (Black/African/Caribbean/Black British), Asian (Asian/Asian British), Mixed ethnic group, Other ethnic group, Unknown and ‘Missing’. Individuals with missing ethnicity data are often at higher risk of worse health outcomes [24]. A further characterisation of the ‘Missing’ ethnic group category was attempted; however, other variables were also unavailable for this group which limited analysis, for example main language was only recorded for 15% (Additional file 2: Tables S1 and S2). To test for heterogeneity within the main ethnic groups, the model was re-run separately for individuals from White, Black, Asian, Mixed and Other backgrounds, this time interacting the variables of interest with the more comprehensive 18 ethnicity categories from the 2011 Census.

Lastly, the ethnicity model is also estimated separately for each delivery mode to assess whether the shift towards telephone and remote consultations is associated with inequalities by ethnic group. Results are presented only for face-to-face consultations and telephone consultations as they comprise 98.5% of total consultations.

A strength of using an ITS model design is that it controls for differences in the consultation rate and trend that may already have existed between the groups pre-pandemic, and therefore the models do not require controls for time-invariant covariates. However, it assumes no changes in the characteristics of the underlying population that could explain observed differences in consultation rates over the study period. The number of individuals registered to a practice declined by 4% between February and December 2020. Temporal trends in the sociodemographic characteristics (age, gender, social deprivation and ethnicity) of the population were investigated. No significant changes were found, indicating that covariate adjustments were not needed (Additional file 3: Figs. S1-S4).

As sensitivity analyses, first the ethnicity models also controlled for age to assess if variations by ethnic group were being confounded by potential differences in age distribution, since age was a major driver of care prioritisation during the pandemic. Second, the multimorbidity model was calibrated for individuals with complex multimorbidity (with three or more LTCs) compared with individuals with only two LTCs. This analysis aimed to explore how results may differ within the heterogeneous multimorbid population.

All analyses were conducted using R version 4.1.2.

## Results

### Descriptives

The sample consisted of 460,084 individuals (13,536,533 person-months), of whom 24% had multimorbidity. The percentage under the age of 40 years was 55%, 32% were between 40 and 59, 11% between 60 and 79, and 2% were 80 years or over. The mean age was 41 years (SD=15), and half of the sample were female (49.8%). For ethnic group, 56% stated they were of White ethnicity (53% within the multimorbid population), 18% (28%) Black/African/Caribbean/Black British, 7% (7%) Asian/Asian British, 5% (5%) Mixed/multiple ethnic groups, 3% (2%) Other, and 2% (1%) Unknown. Ethnicity data was missing for 8% (4%) of the sample. English was considered the main language for 57% of the sample, and 64% lived in socially deprived areas (lowest two quintiles of the national IMD index). The spread of observations was relatively stable across the study period, with 2.6–2.7% of observations in each month. Further characteristics by ethnic group within the multimorbidity population are presented in Additional file 4: Table S1.

Table 1 presents changes in consultation rates by provider type and delivery mode for 4 months in the sample period: February 2018 (the first data point in the sample), February 2020 (the last data point in the pre-pandemic period), April 2020 (the month with the largest fall in consultations during the pandemic) and March 2021 (the last data point in the sample). In the full population, total primary care consultations per 1000 patients fell by 25% in April 2020 when compared to the previous month (399 to 299). The consultation rate experienced an upward trend after April 2020 and there was a 46% growth in March 2021 compared to February 2020 (388 to 566). The mean monthly consultation rate in the pre-pandemic period was 374 per 1000 patients (SD=1013), compared to 436 in the pandemic period (SD=1276), 17% higher. The standard deviation of consultation rates is relatively large as monthly data has more variability and skewness than annual data. In February 2020, face-to-face consultations made up 75% of total consultations, while 24% were telephone. In April 2020, telephone became the most frequent method (61%), while face-to-face dropped to 38%. The shift towards remote consultations was less persistent for nurses, for example in March 2021, 82% of nurse consultations were face-to-face, while it was 45% for GPs and 63% for other healthcare professionals. There were small shifts in the composition of the healthcare providers towards the end of the pandemic period, with the proportion of consultations delivered by nurses decreasing and the proportion by other healthcare professionals increasing. For example, in February 2020, nurses represented 14% and other healthcare professionals represented 17%, but in March 2021 the proportions were 10% and 21% respectively.

**Table 1:** Total primary care consultations by provider type, delivery mode and multimorbidity status

	February 2018		February 2020		April 2020		March 2021	
	Total, %	Consultation rate per 1000 patients	Total, %	Consultation rate per 1000 patients	Total, %	Consultation rate per 1000 patients	Total, %	Consultation rate per 1000 patients
Registered patients	349,800	NA	364,917	NA	362,241	NA	353,233	NA
Total consultations (excluding admin)	123,157	352.1	141,414	387.5	108,424	299.3	199,846	565.8
GP face-to-face	62,504, 50.8%	178.7	69,921, 49.4%	191.6	26,009, 24%	71.8	63,042, 31.5%	178.5
GP telephone	26,540, 21.5%	75.9	25,767, 18.2%	70.6	54,072, 49.9%	149.3	72,704, 36.4%	205.8
GP home	1127, 0.9%	3.2	1120, 0.8%	3.1	354, 0.3%	1.0	528, 0.3%	1.5
GP electronic	88, 0.1%	0.3	121, 0.1%	0.3	384, 0.4%	1.1	2569, 1.3%	7.3
Nurse face-to-face	17,712, 14.4%	50.6	18,842, 13.3%	51.6	6681, 6.2%	18.4	15,811, 7.9%	44.8
Nurse telephone	972, 0.8%	2.8	1193, 0.8%	3.3	5027, 4.6%	13.9	3267, 1.6%	9.2
Nurse home	154, 0.1%	0.4	203, 0.1%	0.6	72, 0.1%	0.2	187, 0.1%	0.5
Nurse electronic	0, 0%	0.0	4, 0%	0.0	3, 0%	0.0	21, 0%	0.1
Other face-to-face	10,004, 8.1%	28.6	17,686, 12.5%	48.5	8877, 8.2%	24.5	26,168, 13.1%	74.1
Other telephone	3929, 3.2%	11.2	6455, 4.6%	17.7	6658, 6.1%	18.4	9665, 4.8%	27.4
Other home	96, 0.1%	0.3	92, 0.1%	0.3	49, 0%	0.1	88, 0%	0.2
Other electronic	31, 0%	0.1	10, 0%	0.0	238, 0.2%	0.7	5796, 2.9%	16.4
Administrative	15,827	45.2	24,876	68.2	18,365	50.7	65,242	184.7
Total consultations for individuals without MM	58,644, 47.6%	223.5	68,997, 48.8%	248.6	46,487, 42.9%	168.9	102,121, 51.1%	379.8
Total consultations for individuals with MM	64,513, 52.4%	738.3	72,417, 51.2%	828.5	61,937, 57.1%	712.4	97,725, 48.9%	1158.5

Within the multimorbid population, total primary care consultations per 1000 patients fell by 18% in April 2020 when compared to the previous month (886 to 712). The consultation rate experienced an upward trend after April 2020 and there was a 40% growth in March 2021 compared to February 2020 (829 to 1159). Individuals of Asian ethnicity had the highest mean consultation rate in the pre-pandemic period (895 per 1000 patients, SD=1517), followed by people from Black (871, SD=1514), Other (806, SD=1458), Mixed (796, SD=1467), White (769, SD=1465), Unknown (758, SD=1463) then Missing (578, SD=1228). However, during the pandemic period the order changed, with individuals of Black ethnicity having the highest rate (1033, SD=1,926), followed by people of Asian (1029, SD=1941), Mixed (981, SD=1881), Other (967, SD=1855), White (907, SD=1811), Unknown (895, SD=1784), and Missing (715, SD=1585). The breakdown by delivery mode and provider type was similar to that of the full population.

### Main results

**Fig. 1:**
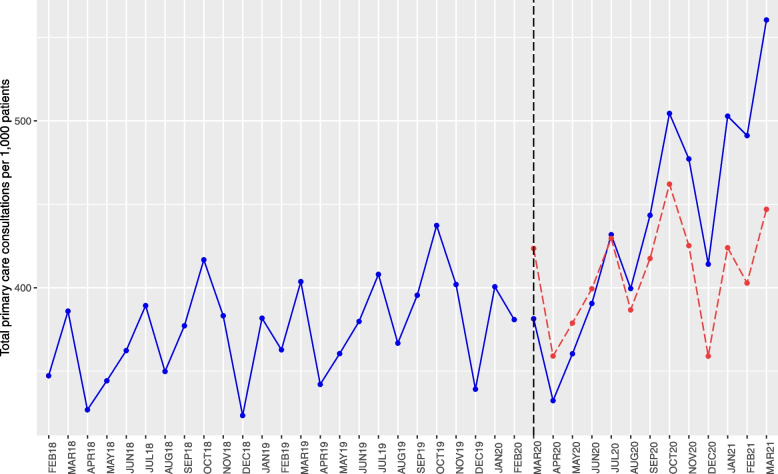
Results of ITS analysis—primary care consultation rates and counterfactual scenario (dashed red line), using full population

In the initial months of the pandemic, overall consultation rates as predicted by the ITS model were lower than expected (Fig. 1). For example, in March 2020 the rates were 10% lower than the counterfactual (381 per 1000 patients, compared to 424). Faster growth in the latter half of the pandemic resulted in consultations then being higher than the counterfactual, for example in March 2021 the rate was 25% higher than what would have occurred without the pandemic (560 vs 447).

Individuals with multimorbidity had 3.6 times (95% CI 3.5–3.6) the rate of consultations compared to those without multimorbidity in the pre-pandemic period (Table 2 and Additional file 5: Tables S1-S3). The pre-pandemic trend was slightly steeper for those with multimorbidity (IRR=1.002 monthly growth rate, 1.002–1.003). During the pandemic, the gap between the two groups grew, with those without multimorbidity experiencing a larger initial fall in primary care consultations from 223 (221–226) consultations per 1000 patients in February 2020 to 208 (205–210) in March 2020, a 7% fall. The rate remained unchanged for those with multimorbidity (879 (869–890) to 882 (870–894), <1% change). Between March 2020 and July 2020, the consultation rate for those with multimorbidity remained over 4 times the rate of those without multimorbidity. For example, in March 2020, the rates were 882 (870–894) and 208 (205–210) consultations per 1000 patients, respectively for each group. Those without multimorbidity then experienced a faster rate of increase in consultations and by March 2021, the differences had reduced to 3.8 times; 1269 (1251–1.285) and 335 consultations (331–339).

**Table 2:** Results of ITS analysis—effect of the pandemic on primary care consultations by multimorbidity status

	Without multimorbidity (baseline)	With multimorbidity	
		Consultation rate	Relative rate^**a**^
**Feb-20**	223.1 (220.6–225.6)	879.4 (868.6–890.3)	3.94
**Mar-20** (Start of Pandemic)	207.8 (205.3–210.3)	881.8 (869.9–893.7)	4.24
**Apr-20**	179.9 (177.8–182)	751.5 (741.8–761.3)	4.18
**May-20**	183.6 (181.5–185.7)	768.6 (759–778.2)	4.19
**Jun-20**	201.7 (199.4–203.9)	816.3 (806.3–826.3)	4.05
**Jul-20**	231.5 (228.9–234)	947.6 (936.3–958.9)	4.09
**Aug-20**	220.2 (217.8–222.7)	854.4 (844.3–864.6)	3.88
**Sep-20**	245.7 (243–248.3)	967 (955.7–978.3)	3.94
**Oct-20**	290.9 (287.7–294.1)	1157.4 (1143.8–1171)	3.98
**Nov-20**	274 (271–277)	1051.1 (1038.5–1063.6)	3.84
**Dec-20**	236.6 (233.9–239.3)	908.2 (897.1–919.3)	3.84
**Jan-21**	289.8 (286.6–293.1)	1165 (1150.5–1179.6)	4.02
**Feb-21**	278.9 (275.7–282.1)	1059.3 (1045.6–1072.9)	3.80
**Mar-21**	334.7 (330.7–338.7)	1268.3 (1251.2–1285.4)	3.79

Estimates are expressed as the consultation rate per 1000 patients, with the 95% confidence interval in parentheses

^a^The relative rate is the consultation rate of individuals with multimorbidity divided by the consultation rate of the model baseline (those without multimorbidity). Number of observations was 12,847,347, after the removal of 1.7% of data points that were identified as outliers (>3.5 studentised residuals). See Supplement 5 for the corresponding figure, and separate figures for face-to-face and telephone consultations

Similar patterns were found when comparing those with complex multimorbidity to those with only two LTCs, with a smaller contraction in primary care consultations for those with complex multimorbidity (Additional file 6: Tables S1 and S2).

**Fig. 2:**
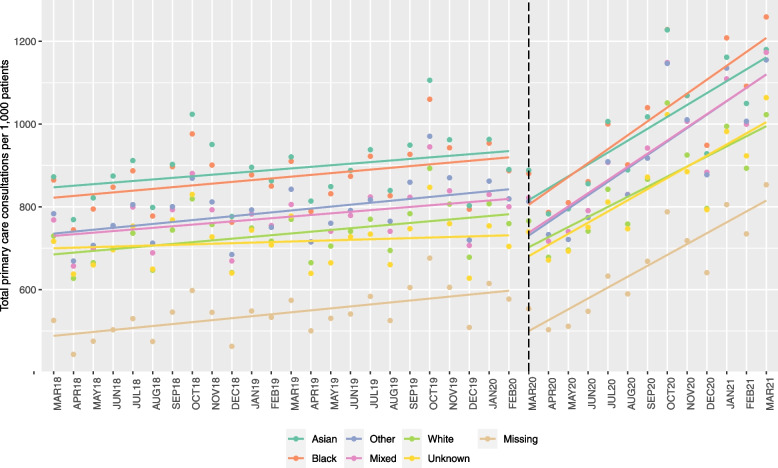
Results of ITS analysis—primary care consultation rates in the multimorbid population, by ethnic group. Notes: For similar figures, but with face-to-face consultations and telephone consultations, please see Supplement 5

Within the multimorbid population, individuals of Asian ethnicity had the highest total consultation rate in the pre-pandemic period (IRR=1.285, 95% CI 1.258–1.312), followed by people of Black (1.204, 1.189–1.219), Other (1.066, 1.030–1.104), Mixed (1.062, 1.035–1.089), White (model baseline) and Unknown (not significantly different to White), then Missing (0.722, 0.702–0.744) ethnicities (Table 3, Fig. 2 and Additional file 5: Tables S1-S3). The clinical drivers of the differences in baseline consultation rates vary by ethnic group. For example, in the dataset, individuals of White ethnicity have a higher rate of anxiety and depression than those of Black ethnicity, while individuals of Black ethnicity experience a higher rate of chronic pain, hypertension and diabetes. The average number of LTCs is slightly higher among people with Black backgrounds; 3.39 (SD=1.6) compared to 3.15 (1.5) for those of Mixed ethnicity for example (Additional file 4: Table S1). There were minor differences in pre-pandemic trends, with individuals of Black (0.999, 0.998–1.000) ethnicity having a slower growth rate in consultations than White ethnicity, while the group with Missing ethnicity had a faster growth rate (1.003, 1.001–1.005). Comparing the consultation rates for each ethnicity to the model baseline (White), between March 2020 and June 2020 the relative rate for those of Black, Asian, Other and Missing ethnic groups decreased slightly compared to White. For example, for individuals with ethnicity Missing, the consultation rate dropped from 577 (95% CI 561–594) per 1000 patients in February 2020 to 554 (534–574) in March 2020 (relative rate dropped from 0.76 times the rate of White to 0.72 times). The rate for people from Asian backgrounds remained a similar proportion to those of White for the rest of the pandemic period, while the rate for the Black, Mixed, Other, Unknown and Missing ethnic groups began growing at faster rates. The highest increase in relative rate was for those of Unknown ethnicity. Between February 2020 and March 2021, the relative rate for people with Unknown ethnicity compared to White went from 0.93 to 1.04; a growth from 704 (674–735) consultations per 1000 patients to 1064 (1006–1121). For those of Mixed and Unknown ethnicities, the relative rate compared to White was higher than the February 2020 baseline throughout the pandemic period.

**Table 3:** Results of ITS analysis—total consultations per 1000 patients by ethnicity (within multimorbid population)

	White—baseline	Black		Asian		Mixed		Other		Unknown		Missing	
		Consultation rate	Relative rate^a^	Consultation rate	Relative rate^a^	Consultation rate	Relative rate^a^	Consultation rate	Relative rate^a^	Consultation rate	Relative rate^a^	Consultation rate	Relative rate^a^
**Feb-20**	759.6 (747.7–771.5)	887.5 (872.4–902.6)	1.17	890.2 (869.4–911.1)	1.17	800.4 (779–821.7)	1.05	819.4 (791.4–847.3)	1.08	704.4 (674–734.9)	0.93	577.2 (560.6–593.9)	0.76
**Mar-20**	766.1 (753.2–778.9)	880.9 (864.4–897.4)	1.15	888.6 (863.3–913.8)	1.16	821.7 (794.6–848.8)	1.07	813.1 (777.9–848.4)	1.06	739.8 (698.8–780.8)	0.97	553.9 (533.8–574.1)	0.72
**Apr-20**	678.1 (667.1–689.1)	786.8 (772.7–800.9)	1.16	783.1 (762.7–803.5)	1.15	717.1 (695.6–738.6)	1.06	733 (704.4–761.6)	1.08	671.5 (638.3–704.8)	0.99	503.2 (486.7–519.7)	0.74
**May-20**	695.8 (684.7–706.8)	810.3 (796.3–824.2)	1.16	795.4 (776.3–814.5)	1.14	740.2 (720–760.4)	1.06	721.3 (696.1–746.5)	1.04	693.3 (662.8–723.8)	1.00	511.4 (496.2–526.5)	0.73
**Jun-20**	741.4 (729.9–753)	860.9 (846.5–875.3)	1.16	855.9 (836.9–874.9)	1.15	790.9 (771.2–810.6)	1.07	774.4 (750–798.7)	1.04	750.7 (721.2–780.2)	1.01	547.7 (533–562.3)	0.74
**Jul-20**	842.5 (829.6–855.5)	1000.4 (984.1–1016.7)	1.19	1006.3 (985.3–1027.3)	1.19	907.6 (886.7–928.4)	1.08	909.1 (883.1–935.1)	1.08	811.6 (783–840.3)	0.96	632.7 (617.2–648.3)	0.75
**Aug-20**	758.5 (746.9–770.1)	901.6 (887–916.1)	1.19	889.4 (871.5–907.2)	1.17	830 (811.9–848.1)	1.09	829.1 (806.9–851.3)	1.09	747.2 (722.8–771.6)	0.99	589.7 (576.1–603.3)	0.78
**Sep-20**	866.5 (853.4–879.6)	1039.6 (1023.1–1056.2)	1.20	1017.6 (997.7–1037.6)	1.17	942.1 (922.1–962.1)	1.09	917.7 (893.9–941.5)	1.06	871.9 (844.3–899.4)	1.01	668.6 (653.7–683.5)	0.77
**Oct-20**	1051.2 (1035.3–1067.1)	1228.4 (1208.9–1248)	1.17	1227.7 (1203.4–1252)	1.17	1148.7 (1124.2–1173.3)	1.09	1146.7 (1116.5–1176.9)	1.09	1022.7 (989.7–1055.7)	0.97	788.1 (770.3–805.8)	0.75
**Nov-20**	925.3 (911.2–939.5)	1068.7 (1051.4–1086.1)	1.15	1069.3 (1047.2–1091.3)	1.16	1006.7 (984–1029.4)	1.09	1010.4 (982.2–1038.7)	1.09	885.1 (854.4–915.7)	0.96	718.4 (701.3–735.5)	0.78
**Dec-20**	796.1 (783.7–808.5)	948.8 (933–964.6)	1.19	928.4 (907.9–948.8)	1.17	883.7 (862.3–905.2)	1.11	877.6 (850.9–904.3)	1.10	793.3 (762.9–823.6)	1.00	641.1 (624.5–657.7)	0.81
**Jan-21**	994.8 (979.1–1010.5)	1208.4 (1187.8–1228.9)	1.21	1161.6 (1134.2–1189)	1.17	1109.3 (1079.9–1138.6)	1.12	1135.4 (1097.1–1173.6)	1.14	982.3 (940.2–1024.4)	0.99	805.4 (782.6–828.2)	0.81
**Feb-21**	893.4 (879–907.9)	1091.5 (1072.2–1110.8)	1.22	1050 (1023–1077)	1.18	999.7 (970.7–1028.8)	1.12	1006.8 (969–1044.6)	1.13	923.4 (878.8–967.9)	1.03	734.7 (711.6–757.8)	0.82
**Mar-21**	1022.9 (1005.6–1040.1)	1259 (1235.5–1282.5)	1.23	1180.1 (1147–1213.2)	1.15	1173.4 (1135.7–1211.1)	1.15	1155.2 (1106.8–1203.6)	1.13	1064 (1006.3–1121.6)	1.04	853.4 (823.6–883.2)	0.83

Estimates are expressed as the consultation rate per 1000 patients, with the 95% confidence interval in parentheses

^a^The relative rate is the consultation rate divided by the consultation rate of the model baseline (White). Number of observations in model was 3,154,547 after the removal of 1.4% of data points that were identified as outliers (>3.5 studentised residuals)

The main results held when models adjusted for age (Additional file 7: Table S1), while suggesting the consultation rate for people of Mixed ethnicity may be slightly underestimated.

There were also inequalities in the change of face-to-face consultations (Table 4 and Additional file 5: Tables S1-S3). For people of Black, Asian and Other ethnicities, the consultation rate fell more relative to White. For example, in April 2020, for individuals of Black ethnicity, the relative rate went from 1.23 times the rate of White ethnicity to 1.19 times (from 657 (645–669) consultations per 1000 patients to 355 (348–362)). For people from Asian backgrounds, the comparable figures were from a rate of 1.26 times to 1.16, and for individuals in the Other ethnicity group it was 1.15 to 1.11. The relative rate began to recover for those of Black and Other ethnicities around December 2020, while the rate remained comparatively low (<1.19 February baseline) for people of Asian ethnicity. The rate for individuals of Mixed ethnicity was similar to White until around July 2020, when the relative rate became higher for those of Mixed ethnicity. For individuals with Unknown ethnicity, the relative rate remained above the February 2020 baseline throughout.

**Table 4:** Results of ITS analysis—face-to-face and telephone consultations per 1000 patients by ethnicity (within multimorbid population)

	White—baseline	Black		Asian		Mixed		Other		Unknown		Missing	
		Consultation rate	Relative rate^a^	Consultation rate	Relative rate^a^	Consultation rate	Relative rate^a^	Consultation rate	Relative rate^a^	Consultation rate	Relative rate^a^	Consultation rate	Relative rate^a^
***Face-to-face consultations***													
**Feb-20**	534.8 (525.7–543.9)	656.7 (644.7–668.8)	1.23	675.9 (659–692.8)	1.26	555.7 (539.8–571.7)	1.04	613.2 (591–635.4)	1.15	490.1 (467.2–513.1)	0.92	422 (408.9–435.2)	0.79
**Mar-20**	357.9 (351.2–364.7)	415.3 (406.5–424.1)	1.16	417.9 (404.2–431.7)	1.17	372.9 (358.5–387.3)	1.04	391.3 (371.5–411.1)	1.09	341.5 (318.7–364.3)	0.95	270.3 (258.5–282.1)	0.76
**Apr-20**	299.1 (293.6–304.5)	354.7 (347.5–361.8)	1.19	348.2 (337.8–358.7)	1.16	313.6 (302.6–324.6)	1.05	332.3 (317.2–347.5)	1.11	282.6 (265.8–299.5)	0.94	226.5 (217.5–235.4)	0.76
**May-20**	305 (299.6–310.4)	356.8 (350–363.7)	1.17	349.4 (339.8–358.9)	1.15	319.9 (309.7–330)	1.05	330 (316.6–343.4)	1.08	292.8 (277.3–308.3)	0.96	232.6 (224.4–240.8)	0.76
**Jun-20**	332.2 (326.5–337.9)	390.2 (383–397.5)	1.17	384.5 (374.8–394.2)	1.16	352 (341.9–362.2)	1.06	363.2 (350–376.5)	1.09	314.2 (299.4–328.9)	0.95	257.9 (249.7–266.1)	0.78
**Jul-20**	386.3 (379.8–392.8)	460.1 (451.8–468.3)	1.19	459.4 (448.6–470.2)	1.19	414.1 (403.1–425)	1.07	431.3 (417.1–445.5)	1.12	364.5 (349.2–379.8)	0.94	301.7 (293–310.4)	0.78
**Aug-20**	356 (350–362)	426.9 (419.3–434.4)	1.20	417 (407.6–426.4)	1.17	381.1 (371.6–390.5)	1.07	400.9 (388.7–413.2)	1.13	332.9 (320.1–345.8)	0.94	278.9 (271.4–286.3)	0.78
**Sep-20**	429.3 (422.2–436.4)	517 (508–526)	1.20	503.2 (492.2–514.2)	1.17	465.8 (454.6–476.9)	1.09	472.4 (458.6–486.2)	1.10	406.8 (391.8–421.8)	0.95	342.5 (333.7–351.3)	0.80
**Oct-20**	534.5 (525.7–543.3)	625.3 (614.5–636.2)	1.17	629.5 (615.7–643.3)	1.18	573.5 (559.7–587.2)	1.07	604.8 (586.9-622.6)	1.13	491.7 (473.3–510)	0.92	410.7 (400.2–421.3)	0.77
**Nov-20**	463.4 (455.6–471.1)	542.9 (533.3–552.6)	1.17	543.4 (531–555.8)	1.17	500.5 (487.9–513.1)	1.08	529.3 (512.8–545.8)	1.14	425.4 (408.4–442.4)	0.92	367.7 (357.7–377.7)	0.79
**Dec-20**	393.9 (387.1–400.6)	471.9 (463.3–480.5)	1.20	464.5 (453.2–475.9)	1.18	434.6 (422.8–446.4)	1.10	452.6 (437.2–468)	1.15	367 (350.8–383.2)	0.93	319.4 (310–328.8)	0.81
**Jan-21**	477.2 (468.9–485.4)	575.6 (564.9–586.4)	1.21	558.7 (544.1–573.4)	1.17	531.5 (515.8–547.3)	1.11	558.5 (537.4–579.5)	1.17	435.9 (414.3–457.5)	0.91	387.4 (374.8–399.9)	0.81
**Feb-21**	442.7 (434.8–450.6)	537.2 (526.8–547.7)	1.21	519.2 (504.4–534)	1.17	487.7 (471.8–503.7)	1.10	507.2 (485.8–528.5)	1.15	434.3 (410–458.6)	0.98	367.4 (354.1–380.6)	0.83
**Mar-21**	528.1 (518.4–537.9)	643.1 (629.9–656.3)	1.22	616.2 (596.9–635.5)	1.17	586.2 (564.9–607.4)	1.11	608.1 (579.5–636.7)	1.15	495.4 (464.2–526.5)	0.94	433.8 (416.3–451.2)	0.82
***Telephone consultations***													
**Feb-20**	189 (184.7–193.3)	187.1 (182.3–191.8)	0.99	179.8 (173.2–186.4)	0.95	207.6 (199.2–215.9)	1.10	176.3 (166.6–186)	0.93	195.8 (183–208.5)	1.04	136.1 (129.8–142.5)	0.72
**Mar-20**	410.9 (401.5–420.4)	488.2 (475.7–500.7)	1.19	498 (479.4–516.6)	1.21	459.6 (440–479.1)	1.12	419.9 (396.4–443.4)	1.02	424.8 (395–454.7)	1.03	287.8 (274.1–301.5)	0.70
**Apr-20**	399.2 (390.1–408.3)	458.5 (447.1–469.9)	1.15	458.3 (442.4–474.2)	1.15	437.5 (420.5–454.6)	1.10	412.1 (391.3–432.9)	1.03	414.3 (388.2–440.4)	1.04	305.3 (292–318.5)	0.76
**May-20**	405 (395.9–414.1)	483.1 (471.4–494.8)	1.19	464.9 (449.8–480)	1.15	448.5 (432.4–464.5)	1.11	422.8 (403.6–442.1)	1.04	396.3 (374–418.6)	0.98	291.8 (280.4–303.3)	0.72
**Jun-20**	424.3 (414.9–433.7)	494.4 (482.7–506.1)	1.17	479.6 (465–494.1)	1.13	461.5 (446.3–476.6)	1.09	437.3 (419.2–455.4)	1.03	454.4 (431.5–477.2)	1.07	305.8 (294.8–316.7)	0.72
**Jul-20**	465.6 (455.4–475.8)	548.7 (535.9–561.4)	1.18	555.4 (539.5–571.4)	1.19	512.5 (496.8–528.2)	1.10	483.8 (465.5–502.1)	1.04	482.1 (460.1–504)	1.04	340.3 (329–351.5)	0.73
**Aug-20**	402.9 (394–411.7)	468.5 (457.6–479.3)	1.16	473.8 (460.6–487.1)	1.18	446.9 (433.8–460.1)	1.11	437.9 (422.2–453.6)	1.09	427.3 (409.2–445.4)	1.06	309.2 (299.5–318.9)	0.77
**Sep-20**	427 (417.7–436.2)	508 (496.4–519.6)	1.19	504.5 (490.8–518.3)	1.18	477.5 (463.8–491.2)	1.12	449 (433.3–464.7)	1.05	456.4 (437.7–475.2)	1.07	324.3 (314.4–334.2)	0.76
**Oct-20**	474.7 (464.4–485)	549.3 (536.8–561.8)	1.16	553.4 (538.1–568.8)	1.17	539.8 (524.1–555.5)	1.14	503.5 (485.7–521.3)	1.06	500.6 (479.6–521.7)	1.05	359 (347.9–370.1)	0.76
**Nov-20**	455.2 (445.3–465.2)	522.2 (510.1–534.3)	1.15	524.8 (509.8–539.8)	1.15	509.4 (494–524.9)	1.12	478.4 (460.5–496.3)	1.05	480 (458.4–501.6)	1.05	341.8 (330.7–352.9)	0.75
**Dec-20**	408.2 (399.1–417.3)	474.1 (462.9–485.4)	1.16	478.5 (464–493)	1.17	464.9 (449.7–480.1)	1.14	448.9 (430.6–467.1)	1.10	438.4 (416.7–460.2)	1.07	319 (307.8–330.3)	0.78
**Jan-21**	495.4 (484.4–506.5)	592.5 (578.3–606.8)	1.20	580 (561.3–598.6)	1.17	563.2 (543.4–583.1)	1.14	540.2 (516.1–564.3)	1.09	522.9 (494–551.9)	1.06	399.3 (384–414.6)	0.81
**Feb-21**	430.9 (421–440.8)	509.1 (496.4–521.8)	1.18	501.5 (484–518.9)	1.16	489.7 (470.8–508.5)	1.14	484.4 (460.4–508.4)	1.12	485.2 (455.1–515.3)	1.13	349.4 (334.6–364.1)	0.81
**Mar-21**	468.4 (457.3–479.4)	560 (545.4–574.5)	1.20	555.4 (534.4–576.3)	1.19	545.1 (522.1–568.2)	1.16	520.6 (492–549.2)	1.11	519.5 (483.3–555.6)	1.11	376.7 (359.1–394.3)	0.80

Estimates are expressed as the consultation rate per 1000 patients, with the 95% confidence interval in parentheses

^a^The relative rate is the consultation rate divided by the consultation rate of the model baseline (White). For the face-to-face model, 1.3% of data points were removed after being identified as outliers (>3.5 studentised residuals). For telephone, 1.4% were removed. Number of observations was 3,157,225 for the face-to-face model and 3,155,350 for the telephone model

For telephone consultations, the rate for individuals of Black, Asian and Other ethnicities increased more than the rate of White (Table 4 and Additional file 5: Tables S1-S3). For example, people from Asian backgrounds had a relative rate compared to White of 0.95 in February 2020 (180 telephone consultations per 1000 patients (95% CI 173–186), but increased to 1.21 in March 2020 (498, 479–517). During the pandemic period the relative rate ranged from 1.15 to 1.21 for this group. For those of Black ethnicity, the baseline was 0.99 in February, and it then ranged from 1.15 to 1.20 during the pandemic period, and for individuals in the Other ethnicity group, it was a baseline of 0.93 and ranged from 1.02 to 1.12. Individuals of Mixed, Unknown and Missing ethnicity categories experienced a rate quite similar to those of White ethnicity, with some increasing slightly more than White towards the end of 2020.

Additional figures for the ITS results can be found in Additional file 8: Figs. S1-S3.

### Heterogeneity within ethnic group

In general, there were no significant differences within each of the five overarching ethnic groups (White, Black, Asian, Mixed, Other) using the more comprehensive 18 ethnic group classification. Four exceptions were noted; individuals of ‘Other Black’ within the Black ethnic group, individuals of ‘Other White’ within the White ethnic group, individuals of Chinese ethnicity within the Asian ethnic group, and ‘Any Other’ in the Other ethnic group had a slightly distinct effect compared to other individuals within their respective ethnic group (Additional file 9: Table S1).

## Discussion

### Summary

This paper documented inequalities in the response to the COVID-19 pandemic on primary care consultations for individuals with multimorbidity. There was an initial decrease in the consultation rate in April 2020, which may be due to patients being deterred from contacting their practices over fears of infection and not wanting to put extra strain on the healthcare system, or patients using alternative services such as NHS 111 and secondary care. Practices may also had reduced capacity amid the initial transition period. Telephone calls were used much more frequently in the pandemic period as primary care providers reduced face-to-face contact in line with NHS England’s recommendation [1]. The initial contraction in consultation rates was smaller for the multimorbid population compared to those without multimorbidity, suggesting that there may have been a successful needs-based prioritisation of multimorbidity at the start of the pandemic. Older people, who have a higher rate of multimorbidity, and those with certain LTCs were classified as ‘vulnerable’ [2]. Patients with the highest risk in this group were actively contacted by the NHS [2] and may have felt more comfortable approaching their practices regularly. After the initial contraction, consultations grew at a faster rate compared to pre-pandemic, likely due to an increase in healthcare need from COVID-19 and deferred appointments from the start of the pandemic. The large increases in consultations from January 2021 may also be partly attributed to the roll-out of the vaccination programme, which commenced in England in December 2020 [25]. In Lambeth specifically, a surge testing that started in February 2021 due to a new COVID-19 variant may partially explain the increase in recorded consultations [26].

Inequalities in the impact of COVID-19 by ethnic group within the multimorbid population were also identified. The pandemic immediately impacted individuals of ethnic minority groups slightly more than those of White ethnicity. However, the primary care consultation rates for people from these ethnic backgrounds recovered quickly, with the exception of individuals of Asian ethnicity. The differences in growth rate meant that while the rate for people of Asian ethnicity had the highest primary care utilisation in the multimorbid population pre-pandemic, utilisation rates for individuals of Black ethnicity became the highest in the pandemic period. The inequalities by delivery mode were stronger, with those of Black, Asian and Other ethnic groups switching from face-to-face to telephone consultations at a higher rate.

### Comparison to existing literature

Previous literature has not reported inequalities in the impact of the pandemic on healthcare utilisation by multimorbidity status and ethnic group, yet some similarities are observed in the overall trends in primary care consultations during the pandemic. An initial contraction in consultations at the start of the pandemic and large shift towards remote delivery is well-documented in the literature [17–19]. Using data from 21 general practices in Bristol, North Somerset and South Gloucestershire Clinical Commissioning Group, Murphy et al. [18] reported a decline in consultations by 17% in April 2020 compared to April 2019 and that 90% and 46% of consultations delivered by GPs and nurses, respectively, were remote. The fall in overall consultations was similar in this study (15%), as was the percentage of remote nurse consultations (43%), yet fewer GP consultations were delivered remotely (67%). The GP practices in our sample may have faced more difficulties in adapting to remote delivery in April 2020. Based on a longer time horizon (up to June 2020), Watt et al. [19] found that remote primary care consultations represented 50–60% of all consultations using data from Clinical Practice Research Datalink, similar to this paper’s findings of 54%.

Existing literature shows that individuals of Black and Asian ethnicities were at a higher risk of severe COVID-19 [9, 12, 13]. This could have two conflicting effects on primary care consultations; to increase primary care utilisation if more healthcare is required, or decrease consultations if the severity of the disease requires hospitalisation rather than primary health care. It is possible the slightly larger initial contraction for individuals of Black ethnicity at the start of the pandemic was partially a result of higher hospitalisation rates from COVID-19, and that the higher growth in consultation rates reflects primary care dealing with a larger COVID-19 and ‘long-COVID’ burden in this population [12, 13]. Analysis of secondary care data during the pandemic is needed to provide further insight into these hypotheses. A similar increase in healthcare use for individuals of Asian ethnicity was not observed, despite this higher risk of COVID-19 for this group. Inequalities in the impact of the COVID-19 response for ethnic minority groups in the UK have been attributed to a range of complex factors, including being poorer, having less suitable housing, type of employment (larger proportion of key workers) and barriers in access to health services [27].

A shift towards remote consultations may also have increased barriers to access for particular groups, including those who lack access to the appropriate technology, those who cannot afford telephone bills or good quality broadband, those who lack private space in their household, those who rely on non-verbal communication, and individuals with low English skills [28, 29]. The larger proportional fall in face-to-face consultations for people with an Asian background may be due to multi-generational, overcrowded households living with someone over the age of 70 and older being more common for individuals of Bangladeshi and Pakistani ethnicity [30]. Having a ‘vulnerable’ person in the household may have deterred these individuals from attending face-to-face consultations. Individuals from Bangladeshi and Pakistani backgrounds are also more likely to have dependent children which may have impeded access to in-person care when school facilities were closed [31]. Face-to-face consultations are typically superior at gathering more patient information, with longer durations and better relationship building compared to telephone and remote consultations [32–34]. A disproportionate reduction in face-to-face consultations may negatively impact the quality of care received by individuals of Asian ethnicity with multimorbidity.

These findings complement current literature on healthcare disruption. Disproportionate impacts of healthcare disruption on health service use and outcomes have been identified among groups defined by age, ethnicity, social deprivation, LTCs and migrant status [35–40]. For example, consequences tend to be magnified among ethnic minority groups [35–37]. Individuals of Black, Mixed and Other ethnicity experienced lower non-COVID-19 hospital use during the lockdown in England, which may indicate a higher risk of unmet healthcare need [35]. This paper finds similar patterns of disruption by ethnic group for primary care utilisation. While there is evidence that the disruption varies by single LTC, none of the previous literature has focused on individuals with multiple LTCs [38]. The variation in disruption by LTC can also help explain some of the ethnic disparities in utilisation, as the clinical drivers for healthcare needs vary by ethnic group. For example, reduction in primary care contacts was largest for diabetic emergencies, which is more prevalent among ethnic minority groups.

Understanding the most common barriers in access to care among ethnic groups is needed to device interventions aimed at reducing inequalities in primary care use and quality. Tailoring and prioritising disease management in a culturally sensitive manner is key to increase trust and promote better relationship building.

### Strengths and limitations

This study expands existing literature looking at the healthcare inequalities associated with the pandemic, focusing on the growing multimorbid population. A large, longitudinal dataset with rich clinical and sociodemographic information was used and the data characterises an urban, deprived, and multi-ethnic borough in London. Ethnic groups overrepresented in this sample were disproportionately affected by the pandemic and the available data on ethnicity allowed for an assessment of related inequalities. Data on ethnicity are often missing in health records impeding inequality analysis [41–44], for example ethnicity was recorded for 78% of patients registered between 2006 and 2012 to the Clinical Practice Research Datalink, up from 27% for those registered 1990–2012 [44]. In contrast, ethnicity data was only missing for 4% of patients in this study’s model by ethnic group. However, this paper only characterised ethnic inequalities in primary care consultations within, but not between, GP practices due to the inclusion of GP practice fixed effects. Further research should also test for the existence of ethnic inequalities between GP practices.

Healthcare utilisation was measured using primary care consultations only and pandemic impacts on secondary care use were not made accessible for this population. This is an important consideration as ethnicity is a risk factor for the severity of COVID-19 symptoms, which will influence whether a patient requires primary or secondary care. The reported increase in total consultations may be a result of telephone consultations being used to assess if an in-person consultation is required, causing a degree of duplication and may overestimate the true healthcare demand. The duration of and rationale for the consultations were not available to understand the extent of this and to evaluate whether differences in the pace of growth between ethnicities reflect changes in actual need or inequalities in accessing care. Misclassification errors in the delivery mode of primary care consultations are possible, particularly for electronic consultations which are more recent and may be harder to identify. However, electronic consultations represent a very small proportion of total primary care consultations. Differences in the vaccine uptake between ethnic groups [45] may also affect variations in healthcare need after the vaccination programme was rolled out in December 2020. However, vaccination status of the patients was not available in the dataset. Multimorbidity was denoted as a binary variable, defined as those with two or more long-term conditions. Sensitivity analyses comparing individuals with complex multimorbidity to those with only two LTCs provided preliminary insights into possible differential pandemic effects within the multimorbid group. The smaller reduction in primary care consultations among the complex multimorbid may suggest that clinical care for this group was particularly prioritised during the pandemic. Further research by counts of LTCs or the most common LTC clusters (which often vary by ethnic group) could reveal more information about pandemic impact and recovery among those with the greatest severity of health concerns. This paper benefited from a high proportion of ethnic minority groups in the data, which allowed for more granular analyses of these groups. However, results may be less generalisable to the wider population. The study population is also younger than the national average, with only 9% aged 65 or over, compared to 19% for England [46]. However, these demographics are typical of many inner-city communities [47]. Lastly, as an ITS approach is quasi-experimental, it has limitations in assigning causality to the pandemic and it is possible that factors not captured by the model influenced the observed trends.

### Conclusions

Primary care utilisation of individuals with multimorbidity was significantly affected by the COVID-19 pandemic. While there is evidence of a successful needs-based prioritisation of multimorbidity patients within primary care at the start of the pandemic, inequalities among ethnic minority groups were found.

Understanding inequalities generated by the response to the COVID-19 pandemic is essential to identify those who may have under-used primary care. These inequalities may increase their risk of future health complications if their conditions were less frequently monitored or new diagnoses delayed. Further research into the consequences of possible late or missed diagnosis during the pandemic, in particular for the disadvantaged groups identified in this paper, should be considered. As this study explores primary care consultations only, future work is required into secondary and tertiary care usage to identify a more holistic view of the effect of the COVID-19 pandemic on healthcare utilisation of individuals with multimorbidity.

## Supplementary Information

**Additional file 1: Table S1.** List of 32 long-term conditions.

**Additional file 2: **Missing Ethnicity Data Analysis. **Table S1.** Analysis of top 10 languages. **Table S2.** Analysis of top 10 countries of birth (COBs).

**Additional file 3: **Temporal trends in covariates, February 2018 to March 2021. **Figure S1.** Proportion of females. **Figure S2.** Proportion by age groups. **Figure S3.** Proportion by ethnic group. Figure S4. Proportion by social deprivation (IMD).

**Additional file 4: Table S1.** Summary of characteristics by ethnic group, within the multimorbidity population.

**Additional file 5: **Parameter estimates from the ITS models. **Table S1.** Results of ITS analysis - Effect of pandemic on primary care consultations by multimorbidity status. **Table S2.** Results of ITS analysis - Effect of pandemic on total primary care consultations within multimorbid population, by ethnicity. **Table S3.** Results of ITS analysis - Effect of pandemic on face-to-face and telephone consultations within multimorbid population, by ethnicity.

**Additional file 6: **Results of ITS analysis - Effect of the pandemic on primary care consultations by complex multimorbidity status (Sensitivity analysis). **Table S1.** Results of ITS  analysis - Effect of pandemic on total consultations, by complex multimorbidity status. **Table S2.** Results of ITS analysis - Effect of pandemic on total consultations, by complex multimorbidity status.

**Additional file 7: Table S1.** Results of ITS analysis - Effect of the pandemic on primary care consultations by ethnic group, while controlling for age (Sensitivity analysis).

**Additional file 8: **Figures of ITS model results. **Figure S1.** Total consultations by multimorbidity status. **Figure S2.** Face-to-Face consultations by multimorbidity status. **Figure S3.** Telephone consultations by multimorbidity status. **Figure S4.** Face-to-Face consultations by ethnic group. **Figure S5.** Telephone consultations by ethnic group.

**Additional file 9: **Results of ITS analysis - Effect of pandemic on primary care consultations by 18-category ethnic groups. **Table S1.** Results of ITS analysis - Effect of pandemic on total consultations, within the multimorbidity population – Heterogeneity within ethnic group.

## Data Availability

The datasets generated and/or analysed during the current study are not publicly available due to the condition agreed by included general practices for pseudonymised data extraction that the data would not be shared beyond parties named in the Data Sharing Agreement, but limited datasets from the corresponding author on reasonable request.

## Abbreviations

GP: General practitioner
IMD: Index of multiple deprivation
IRR: Incidence rate ratio
ITS: Interrupted time series
LTC: Long-term condition
NHS: The National Health Service
UK: United Kingdom
