# Supplementary material for: Ethnic inequalities in the impact of COVID-19 on primary care consultations: a time series analysis of 460,084 individuals with multimorbidity in South London
Source: BMC Med. 2023 Jan 19;21:26. doi: 10.1186/s12916-022-02720-7 (PMC9851584; doi:10.1186/s12916-022-02720-7)
Supplement: Supplementary file 1 — Additional file 1: Table S1. List of 32 long-term conditions. [file 12916_2022_2720_MOESM1_ESM.docx]

**Additional File 1 – Table S1. List of 32 long-term conditions**

Multimorbidity was defined as having two or more of the 32 long-term conditions listed in the following table. Conditions were selected to reflect demographic and morbidity patterns in an inner-city context [19].

| Alcohol Dependence | Inflammatory Bowel Disease (IBD) |
| --- | --- |
| Atrial Fibrillation | Learning Disabilities |
| Anxiety Disorders | Liver Disease |
| Asthma | Lupus |
| Cancer | Serious Mental Illness (SMI) |
| Coronary Heart Disease (CHD) (includes Angina, MI) | Morbid Obesity (BMI ≥40) |
| Chronic Kidney Disease (CKD) - Stages 3-5 | Multiple Sclerosis |
| Chronic obstructive pulmonary disease (COPD) | Osteoporosis |
| Chronic Pain | Osteoarthritis |
| Dementia (includes Alzheimer's) | Peripheral Arterial Disease (PAD)/Peripheral Vascular Disease (PVD) |
| Depression | Parkinson's disease |
| Diabetes mellitus | Rheumatoid Arthritis |
| Epilepsy | Sickle-Cell Anaemia |
| Heart Failure | Substance dependency |
| HIV/AIDS | Stroke and Transient Ischemic Attack (TIA) |
| Hypertension | Viral Hepatitis (B & C) |
