# Supplementary material for: Ethnic inequalities in the impact of COVID-19 on primary care consultations: a time series analysis of 460,084 individuals with multimorbidity in South London
Source: BMC Med. 2023 Jan 19;21:26. doi: 10.1186/s12916-022-02720-7 (PMC9851584; doi:10.1186/s12916-022-02720-7)
Supplement: Supplementary file 2 — Additional file 2: Missing Ethnicity Data Analysis. Table S1. Analysis of top 10 languages. Table S2. Analysis of top 10 countries of birth (COBs). [file 12916_2022_2720_MOESM2_ESM.docx]

**Additional File 2 - Missing Ethnicity Data Analysis**

Additional analysis was carried out on individuals with ‘Missing’ ethnicity data to better understand the composition of this group. Language and country of birth (COB) were analysed (**Table S1** and **S2** below), however, these variables were also frequently missing for this group. Only 15% and 10% had language and COB data available, respectively, which limits the interpretation of the analysis. From the data available, individuals with missing ethnicity data were less likely to be born in the United Kingdom and to have English as their first language.

**Table S1. Analysis of top 10 languages**

| **COB** | **Population with ‘Ethnicity’ Missing (%)** | **Remaining Population (%)** | **Difference (%)** |
| --- | --- | --- | --- |
| English | 55.9 | 74.0 | -18.1 |
| Spanish | 12.7 | 5.3 | 7.4 |
| Portuguese | 10.3 | 4.8 | 5.5 |
| Italian | 3.4 | 2.0 | 1.4 |
| French | 3.2 | 2.2 | 1.0 |
| Polish | 2.0 | 2.0 | 0.0 |
| Arabic | 1.0 | 0.8 | 0.2 |
| Romanian | 1.0 | 0.4 | 0.6 |
| Somali | 0.9 | 0.8 | 0.1 |
| German | 0.9 | 0.5 | 0.4 |
| *% of population with language available* | *14.7* | *82.4* | *-67.7* |

**Table S2. Analysis of top 10 countries of birth (COBs)**

| **COB** | **Population with ‘Ethnicity’ Missing (%)** | **Remaining Population (%)** | **Difference (%)** |
| --- | --- | --- | --- |
| United Kingdom | 25.7 | 45.3 | -19.6 |
| Portugal | 7.6 | 3.8 | 3.8 |
| Spain | 4.8 | 2.0 | 2.8 |
| Italy | 4.3 | 2.5 | 1.8 |
| Brazil | 4.0 | 1.7 | 2.3 |
| France | 3.0 | 1.7 | 1.3 |
| Australia | 2.5 | 1.8 | 0.7 |
| Ecuador | 2.5 | 1.3 | 1.1 |
| Columbia | 2.3 | 1.5 | 0.8 |
| Poland | 1.9 | 2.5 | -0.5 |
| *% of population with COB available* | *9.7* | *54.0* | *-44.3* |
