# Supplementary material for: Ethnic inequalities in the impact of COVID-19 on primary care consultations: a time series analysis of 460,084 individuals with multimorbidity in South London
Source: BMC Med. 2023 Jan 19;21:26. doi: 10.1186/s12916-022-02720-7 (PMC9851584; doi:10.1186/s12916-022-02720-7)
Supplement: Supplementary file 3 — Additional file 3: Temporal trends in covariates, February 2018 to March 2021. Figure S1. Proportion of females. Figure S2. Proportion by age groups. Figure S3. Proportion by ethnic group. Figure S4. Proportion by social deprivation (IMD). [file 12916_2022_2720_MOESM3_ESM.docx]

**Additional File 3 – Temporal trends in covariates, February 2018 to March 2021**

Temporal trends in the sociodemographic characteristics (age, gender, social deprivation, and ethnicity) of the population were investigated. No significant changes were found, indicating that covariate adjustments were not needed.

**Figure S1. Proportion of females**

**
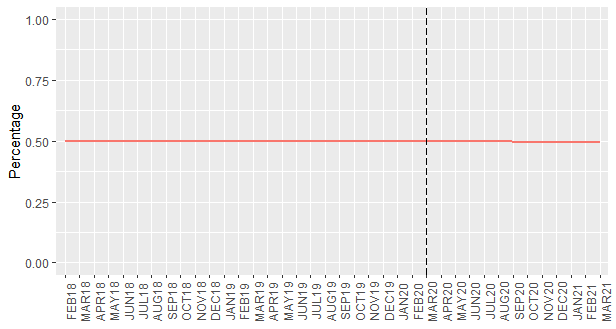
**

**Figure S2. Proportion by age groups**

**
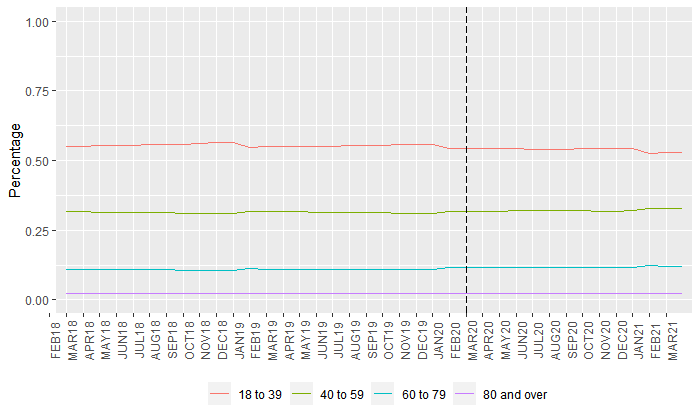
**

**Figure S3. Proportion by ethnic group**

**
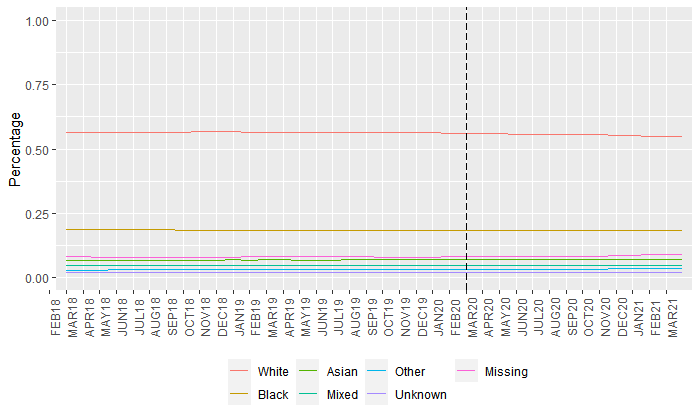
**

**Figure S4. Proportion by social deprivation (IMD)**

**
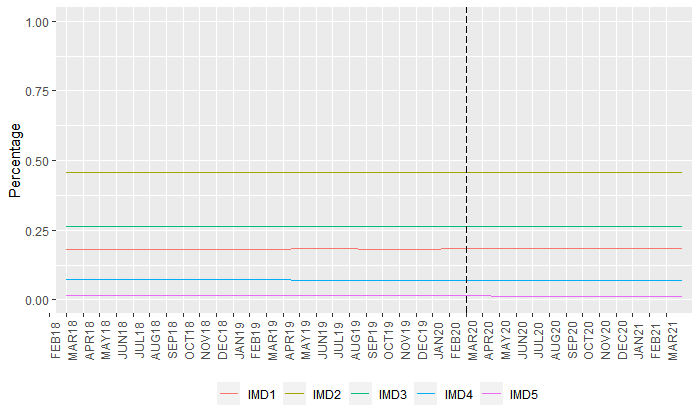
**

Note: IMD 1 = Most deprived, IMD 5 = least deprived
