# Supplementary material for: Ethnic inequalities in the impact of COVID-19 on primary care consultations: a time series analysis of 460,084 individuals with multimorbidity in South London
Source: BMC Med. 2023 Jan 19;21:26. doi: 10.1186/s12916-022-02720-7 (PMC9851584; doi:10.1186/s12916-022-02720-7)
Supplement: Supplementary file 4 — Additional file 4: Table S1. Summary of characteristics by ethnic group, within the multimorbidity population. [file 12916_2022_2720_MOESM4_ESM.docx]

**Additional File 4 – Table S1. Summary of characteristics by ethnic group, within the multimorbidity population**

|  | **White** | **Black** | **Asian** | **Mixed** | **Other** | **Unknown** | **Missing** |
| --- | --- | --- | --- | --- | --- | --- | --- |
| Number of observations with multimorbidity | 1,734,601 | 914,617 | 226,044 | 155,884 | 82,136 | 49,164 | 139,922 |
| Multimorbidity prevalence (% of total population) | 22.84% | 36.64% | 23.82% | 23.88% | 18.40% | 17.57% | 12.50% |
| Mean Age in years (SD) | 53.10 (17.59) | 55.28 (16.03) | 56.53 (16.92) | 47.24 (16.92) | 52.37 (15.09) | 50.37 (17.19) | 49.29 (17.22) |
| Proportion of Males | 46.30% | 39.67% | 47.30% | 38.29% | 44.07% | 50.12% | 49.99% |
| Proportion in IMD 1 | 18.25% | 30.29% | 15.88% | 23.65% | 23.57% | 19.58% | 22.72% |
| Proportion in IMD 2 | 44.63% | 48.62% | 43.14% | 47.05% | 50.30% | 43.14% | 44.63% |
| Proportion in IMD 3 | 27.54% | 17.41% | 28.75% | 23.27% | 21.31% | 29.02% | 25.16% |
| Proportion in IMD 4 | 7.17% | 2.74% | 10.37% | 4.16% | 3.46% | 6.68% | 5.70% |
| Proportion in IMD 5 | 1.27% | 0.23% | 1.15% | 0.81% | 0.38% | 1.05% | 0.91% |
| Average number of LTCs (SD) | 3.33 (1.65) | 3.39 (1.58) | 3.36 (1.63) | 3.15 (1.44) | 3.18 (1.44) | 3.13 (1.50) | 2.89 (1.24) |
| Top 5 most prevalent LTCs | Chronic Pain (55.20%) | Chronic Pain (61.82%) | Chronic Pain (58.93%) | Chronic Pain (55.66%) | Chronic Pain (61.24%) | Chronic Pain (53.32%) | Chronic Pain (52.05%) |
|  | Anxiety  (53.32%) | Hypertension (50.76%) | Hypertension (45.22%) | Anxiety  (51.20%) | Anxiety  (49.24%) | Anxiety  (49.35%) | Anxiety  (50.15%) |
|  | Depression (48.06%) | Anxiety  (36.98%) | Diabetes  (38.96%) | Depression (46.94%) | Depression (43.87%) | Depression (44.67%) | Depression (41.44%) |
|  | Hypertension (28.97%) | Depression (30.68%) | Anxiety  (38.95%) | Hypertension (28.25%) | Hypertension (32.17%) | Hypertension (30.00%) | Hypertension (26.41%) |
|  | Asthma  (24.38%) | Diabetes  (28.39%) | Depression (31.24%) | Asthma  (25.84%) | Diabetes  (24.18%) | Asthma  (23.26%) | Asthma  (22.35%) |

a
