# Supplementary material for: Ethnic inequalities in the impact of COVID-19 on primary care consultations: a time series analysis of 460,084 individuals with multimorbidity in South London
Source: BMC Med. 2023 Jan 19;21:26. doi: 10.1186/s12916-022-02720-7 (PMC9851584; doi:10.1186/s12916-022-02720-7)
Supplement: Supplementary file 5 — Additional file 5: Parameter estimates from the ITS models. Table S1. Results of ITS analysis - Effect of pandemic on primary care consultations by multimorbidity status. Table S2. Results of ITS analysis - Effect of pandemic on total primary care consultations within multimorbid population, by ethnicity. Table S3. Results of ITS analysis - Effect of pandemic on face-to-face and telephone consultations within multimorbid population, by ethnicity. [file 12916_2022_2720_MOESM5_ESM.docx]

**Additional File 5 – Parameter estimates from the ITS models**

The results for the ITS models are presented below. **Table S1** contains the total consultations analysis by multimorbidity status, **Table S2** is total consultations by ethnic group, and **Table S3** contains face-to-face and telephone by ethnic group.

**Table S1. Results of ITS analysis - Effect of pandemic on primary care consultations by multimorbidity status**

|  | **(1) Without Multimorbidity (Baseline)** | **(2) With Multimorbidity** |
| --- | --- | --- |
| **Pre-pandemic baseline at time=0** | 0.169*** | 3.557*** |
|  | (0.001) | (0.015) |
| **Pre-pandemic trend (per month)** | 1.004*** | 1.002*** |
|  | (0.000) | (0.000) |
| **Change in level after pandemic (immediate effect)** | 0.347*** | 1.242*** |
|  | (0.005) | (0.029) |
| **Change in slope after pandemic (gradual effect, per month)** | 1.036*** | 0.994*** |
|  | (0.000) | (0.001) |
| **Number of observations** | 9,752,942 | 3,094,405 |

The dependent variable is total consultations. The coefficients for those without Multimorbidity can be interpreted as follows: 0.169 is the intercept at February 2018, 1.004 is the growth rate per month pre-pandemic, 0.347 is the relative change in level for the pandemic period meaning the intercept of the new trendline is 0.347*0.169 (=0.059), and 1.036 is the relative change in trend giving a pandemic monthly growth rate of 1.036*1.004 (=1.040). Estimates for those with multimorbidity are expressed as IRRs relative to the estimates for those without multimorbidity (the baseline). Number of observations was 12,847,347, after the removal of 1.7% of data points that were identified as outliers (>3.5 studentised residuals). Standard errors in parenthesises: ***p-value <0.001, **p-value<0.01, *p-value<0.05.

**Table S2. Results of ITS analysis - Effect of pandemic on total primary care consultations within multimorbid population, by ethnicity**

|  | **White (Baseline)** | **Black** | **Asian** | **Mixed** | **Other** | **Unknown** | **Missing** |
| --- | --- | --- | --- | --- | --- | --- | --- |
| **Pre-pandemic baseline at time=0** | 0.538*** | 1.204*** | 1.285*** | 1.062*** | 1.066*** | 1.018 | 0.722*** |
|  | (0.004) | (0.008) | (0.014) | (0.014) | (0.019) | (0.023) | (0.011) |
| **Pre-pandemic trend (per month)** | 1.005*** | 0.999* | 0.999 | 1.000 | 1.001 | 0.997 | 1.003** |
|  | (0.000) | (0.000) | (0.001) | (0.001) | (0.001) | (0.002) | (0.001) |
| **Change in level after pandemic (immediate effect)** | 0.543*** | 0.885*** | 0.954 | 0.866* | 0.850 | 0.905 | 0.777*** |
|  | (0.012) | (0.030) | (0.056) | (0.061) | (0.080) | (0.112) | (0.061) |
| **Change in slope after pandemic (gradual effect, per month)** | 1.022*** | 1.005*** | 1.001 | 1.006* | 1.005 | 1.006 | 1.008** |
|  | (0.001) | (0.001) | (0.002) | (0.002) | (0.003) | (0.004) | (0.003) |
| **Number of observations** | 1,656,511 | 873,998 | 215,878 | 148,723 | 78,411 | 46,896 | 134,130 |

The coefficients for the White ethnicity can be interpreted as follows: 0.538 is the intercept at February 2018, 1.005 is the growth rate per month pre-pandemic, 0.543 is the relative change in level for the pandemic period meaning the intercept of the new trendline is 0.538*0.543 (=0.292), and 1.022 is the relative change in trend giving a pandemic monthly growth rate of 1.022*1.005 (=1.027). Estimates for Black, Asian, Mixed, Other, Unknown and Missing are expressed as IRRs relative to White (the baseline). Model only included the multimorbid population, with 3,154,547 observations, after 1.4% of data points were removed when identified as outliers (>3.5 studentised residuals). Standard errors in parenthesises: ***p-value <0.001, **p-value<0.01, *p-value<0.05.

**Table S3. Results of ITS analysis - Effect of pandemic on face-to-face and telephone consultations within Multimorbid population, by ethnicity**

|  | **White (Baseline)** | **Black** | **Asian** | **Mixed** | **Other** | **Unknown** | **Missing** |
| --- | --- | --- | --- | --- | --- | --- | --- |
| ***Face-to-face*** |  |  |  |  |  |  |  |
| **Pre-pandemic baseline at time=0** | 0.268*** | 1.261*** | 1.342*** | 1.064*** | 1.135*** | 0.997 | 0.721*** |
|  | (0.002) | (0.008) | (0.015) | (0.015) | (0.021) | (0.025) | (0.012) |
| **Pre-pandemic trend (per month)** | 1.006*** | 0.999** | 0.999 | 0.999 | 1.000 | 0.998 | 1.004*** |
|  | (0.000) | (0.000) | (0.001) | (0.001) | (0.001) | (0.002) | (0.001) |
| **Change in level after pandemic (immediate effect)** | 0.289*** | 0.862*** | 0.875 | 0.859 | 0.843 | 1.036 | 0.834 |
|  | (0.007) | (0.034) | (0.06) | (0.071) | (0.092) | (0.152) | (0.078) |
| **Change in slope after pandemic (gradual effect, per month)** | 1.029*** | 1.004** | 1.002 | 1.006* | 1.005 | 1.000 | 1.005 |
|  | (0.001) | (0.001) | (0.002) | (0.003) | (0.004) | (0.005) | (0.003) |
| ***Telephone*** |  |  |  |  |  |  |  |
| **Pre-pandemic baseline at time=0** | 0.271*** | 1.034*** | 1.096*** | 1.056** | 0.897*** | 1.150*** | 0.728*** |
|  | (0.003) | (0.01) | (0.019) | (0.021) | (0.026) | (0.039) | (0.017) |
| **Pre-pandemic trend (per month)** | 1.001* | 1.000 | 0.998* | 1.002 | 1.001 | 0.993** | 1.003 |
|  | (0.000) | (0.001) | (0.001) | (0.001) | (0.002) | (0.002) | (0.002) |
| **Change in level after pandemic (immediate effect)** | 1.535*** | 1.166*** | 1.122 | 0.909 | 1.006 | 0.698* | 0.849 |
|  | (0.044) | (0.051) | (0.085) | (0.082) | (0.123) | (0.109) | (0.087) |
| **Change in slope after pandemic (gradual effect, per month)** | 1.013*** | 1.001 | 1.005 | 1.003 | 1.005 | 1.014* | 1.005 |
|  | (0.001) | (0.002) | (0.003) | (0.003) | (0.004) | (0.006) | (0.004) |

Estimates for Black, Asian, Mixed and Other are expressed as IRRs relative to White (the baseline). Model only included the multimorbid population, with 3,157,225 observations for the face-to-face model, after 1.3% of data points were removed as outliers (>3.5 studentised residuals). The telephone model contained 3,155,350 observations, after 1.4% of data points were removed for outliers. Standard errors in parenthesises: ***p-value <0.001, **p-value<0.01, *p-value<0.05.
