# Supplementary material for: Ethnic inequalities in the impact of COVID-19 on primary care consultations: a time series analysis of 460,084 individuals with multimorbidity in South London
Source: BMC Med. 2023 Jan 19;21:26. doi: 10.1186/s12916-022-02720-7 (PMC9851584; doi:10.1186/s12916-022-02720-7)
Supplement: Supplementary file 6 — Additional file 6: Results of ITS analysis - Effect of the pandemic on primary care consultations by complex multimorbidity status (Sensitivity analysis). Table S1. Results of ITS analysis - Effect of pandemic on total consultations, by complex multimorbidity status. Table S2. Results of ITS analysis - Effect of pandemic on total consultations, by complex multimorbidity status. [file 12916_2022_2720_MOESM6_ESM.docx]

**Additional File 6 –** **Results of ITS analysis - Effect of the pandemic on primary care consultations by complex multimorbidity status (Sensitivity analysis)**

To explore heterogeneity within the multimorbidity population, the main model estimating the impact of the pandemic on total primary care consultations of individuals was rerun to compare those with complex multimorbidity - with three or more long-term conditions (LTCs) - to individuals with only two LTCs. **Table S1** is the ITS model output while **Table S2** is the predicted absolute consultations rates from the model.

**Table S1. Results of ITS analysis - Effect of pandemic on total consultations, by complex multimorbidity status**

|  | **(1) Without Complex Multimorbidity (Baseline)** | **(2) With Complex Multimorbidity** |
| --- | --- | --- |
| **Pre-pandemic baseline at time=0** | 0.386*** | 1.972*** |
|  | (0.003) | (0.012) |
| **Pre-pandemic trend (per month)** | 1.005*** | 0.999** |
|  | (0.000) | (0.000) |
| **Change in level after pandemic (immediate effect)** | 0.434*** | 1.258*** |
|  | (0.012) | (0.041) |
| **Change in slope after pandemic (gradual effect, per month)** | 1.029*** | 0.993*** |
|  | (0.001) | (0.001) |
| **Number of observations** | 1,729,891 (41.4%) | 2,446,534 (58.6%) |

Standard errors in parenthesises. ***p-value <0.001, **p-value<0.01, *p-value<0.05, **∙** p-value<0.1. Estimates are expressed as IRRs relative the baseline (those with 2 LTCs). Complex multimorbidity is defined has having 3 or more LTCs. Model only includes the multimorbid population.

**Table S2. Results of ITS analysis Effect of pandemic on total consultations, by complex multimorbidity status**

|  | **Without Complex Multimorbidity** | **Complex Multimorbidity** | |
| --- | --- | --- | --- |
|  |  | **Consultation Rate** | **Relative Rate*** |
| Feb-20 | 511.7 (503.4-520) | 992.6 (977.4-1007.8) | 1.94 |
| Mar-20 | 491.6 (482.8-500.4) | 1017.5 (1001.1-1034) | 2.07 |
| Apr-20 | 440.8 (433.2-448.3) | 896.5 (882.4-910.7) | 2.03 |
| May-20 | 452.5 (445-460) | 919.1 (904.9-933.4) | 2.03 |
| Jun-20 | 488.3 (480.5-496.1) | 975.4 (960.5-990.4) | 2.00 |
| Jul-20 | 559.6 (550.8-568.4) | 1116.7 (1099.8-1133.6) | 2.00 |
| Aug-20 | 513.8 (505.8-521.8) | 1002.9 (987.7-1018) | 1.95 |
| Sep-20 | 587.9 (578.8-596.9) | 1145 (1127.9-1162.1) | 1.95 |
| Oct-20 | 704 (693.3-714.8) | 1374.7 (1354.1-1395.2) | 1.95 |
| Nov-20 | 634.8 (624.9-644.7) | 1207.1 (1188.9-1225.4) | 1.90 |
| Dec-20 | 552.6 (543.8-561.4) | 1050 (1033.8-1066.1) | 1.90 |
| Jan-21 | 691.9 (680.7-703.2) | 1307.6 (1287.5-1327.7) | 1.89 |
| Feb-21 | 634.1 (623.4-644.8) | 1187.8 (1169.1-1206.4) | 1.87 |
| Mar-21 | 734 (721-747) | 1347.9 (1326-1369.8) | 1.84 |

Estimates are expressed as the consultation rate per 1,000 patients, with the 95% confidence interval is in parentheses. *The relative rate for complex multimorbidity is the consultation rate for those with complex multimorbidity divided by the consultation rate for those with only 2 LTCs (baseline).
