# Supplementary material for: Ethnic inequalities in the impact of COVID-19 on primary care consultations: a time series analysis of 460,084 individuals with multimorbidity in South London
Source: BMC Med. 2023 Jan 19;21:26. doi: 10.1186/s12916-022-02720-7 (PMC9851584; doi:10.1186/s12916-022-02720-7)
Supplement: Supplementary file 7 — Additional file 7: Table S1. Results of ITS analysis - Effect of the pandemic on primary care consultations by ethnic group, while controlling for age (Sensitivity analysis). [file 12916_2022_2720_MOESM7_ESM.docx]

**Additional File 7 – Table S1: Results of ITS analysis - Effect of the pandemic on primary care consultations by ethnic group, while controlling for age (Sensitivity analysis)**

The three ethnicity models in the main specification were rerun to control for age, to assess the impact of potential differences in the age distribution across ethnic groups. The dependent variables were total consultations, face-to-face consultations and telephone consultations, and included the multimorbidity population only. Only the estimates for the variables of interest (pandemic parameters) have been presented.

|  | **White (Baseline)** | **Black** | **Asian** | **Mixed** | **Other** | **Unknown** | **Missing** |
| --- | --- | --- | --- | --- | --- | --- | --- |
| ***Total Consultations*** |  |  |  |  |  |  |  |
| **Change in level after pandemic (immediate effect)** | 0.534*** | 0.904** | 0.974 | 0.874 | 0.880 | 0.920 | 0.792** |
|  | (0.012) | (0.032) | (0.060) | (0.064) | (0.086) | (0.117) | (0.064) |
| **Change in slope after pandemic (gradual effect, per month)** | 1.022*** | 1.004*** | 1.000 | 1.006* | 1.004 | 1.005 | 1.007** |
|  | (0.001) | (0.001) | (0.002) | (0.002) | (0.003) | (0.004) | (0.003) |
| ***Face-to-Face*** |  |  |  |  |  |  |  |
| **Change in level after pandemic (immediate effect)** | 0.286*** | 0.871*** | 0.883 | 0.858 | 0.863 | 1.033 | 0.841 |
|  | (0.008) | 0.036 | 0.063 | 0.074 | 0.098 | 0.159 | 0.083 |
| **Change in slope after pandemic (gradual effect, per month)** | 1.029*** | 1.004** | 1.002 | 1.006* | 1.004 | 1.000 | 1.004 |
|  | (0.001) | (0.001) | (0.002) | (0.003) | (0.004) | (0.005) | (0.003) |
| ***Telephone*** |  |  |  |  |  |  |  |
| **Change in level after pandemic (immediate effect)** | 1.507*** | 1.183*** | 1.139 | 0.915 | 1.026 | 0.712* | 0.862 |
|  | (0.046) | (0.055) | (0.092) | (0.087) | (0.133) | (0.118) | (0.094) |
| **Change in slope after pandemic (gradual effect, per month)** | 1.013*** | 1.000 | 1.004 | 1.003 | 1.004 | 1.013* | 1.005 |
|  | (0.001) | (0.002) | (0.003) | (0.003) | (0.005) | (0.006) | (0.004) |

Standard errors in parenthesises. ***p-value <0.001, **p-value<0.01, *p-value<0.05, **∙** p-value<0.1. Estimates are expressed as IRRs relative the baseline (White). Models only included the multimorbid population
