# Supplementary material for: Ethnic inequalities in the impact of COVID-19 on primary care consultations: a time series analysis of 460,084 individuals with multimorbidity in South London
Source: BMC Med. 2023 Jan 19;21:26. doi: 10.1186/s12916-022-02720-7 (PMC9851584; doi:10.1186/s12916-022-02720-7)

**Additional File 8 – Figures of ITS model results**

Figures S1 to S3 present the ITS results by multimorbidity status for total consultations, face-to-face consultations and telephone consultations. The consultation rate for those with multimorbidity is plotted on the primary axis, while the rate for those without is on the secondary axis. Figures D and E present the results by ethnic group for face-to-face and telephone. The results for the total consultations by ethnic group is in the main specification. The dotted vertical line on all figures represents the start of the pandemic.

**Figure S1. Total consultations by multimorbidity status**


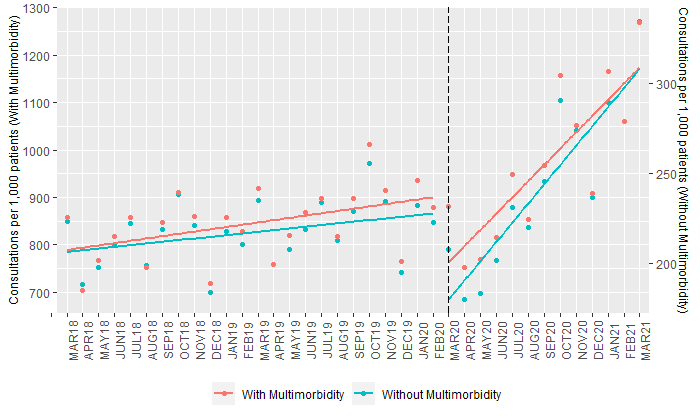


**Figure S2. Face-to-Face consultations by multimorbidity status
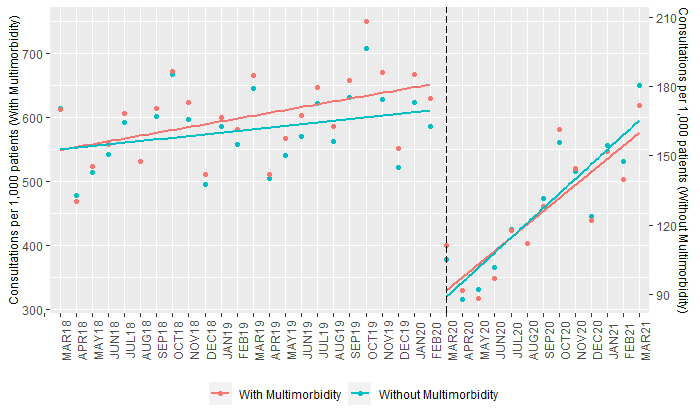
**


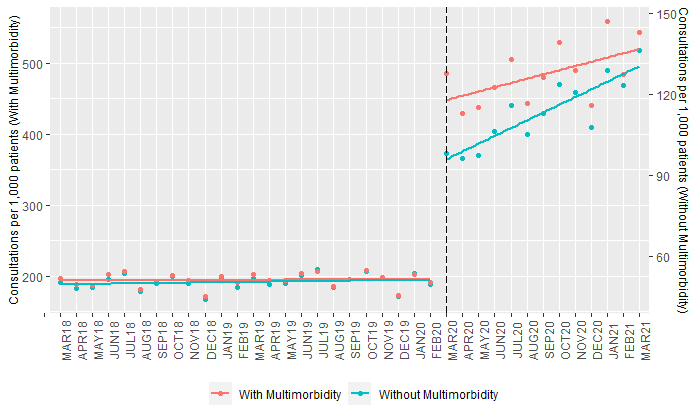
**Figure S3. Telephone consultations by multimorbidity status**

**Figure S4. Face-to-Face consultations by ethnic group**


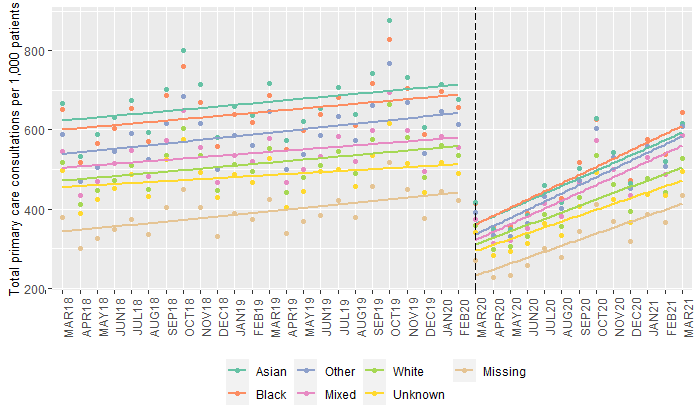


**Figure S5. Telephone consultations by ethnic group**


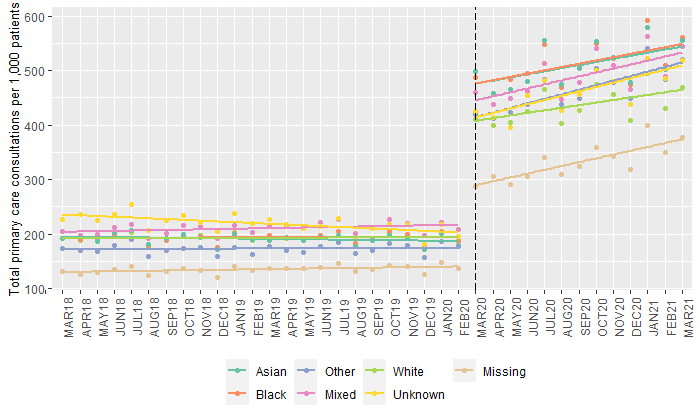

Supplement: Supplementary file 8 — Additional file 8: Figures of ITS model results. Figure S1. Total consultations by multimorbidity status. Figure S2. Face-to-Face consultations by multimorbidity status. Figure S3. Telephone consultations by multimorbidity status. Figure S4. Face-to-Face consultations by ethnic group. Figure S5. Telephone consultations by ethnic group. [file 12916_2022_2720_MOESM8_ESM.docx]
