# Supplementary material for: Ethnic inequalities in the impact of COVID-19 on primary care consultations: a time series analysis of 460,084 individuals with multimorbidity in South London
Source: BMC Med. 2023 Jan 19;21:26. doi: 10.1186/s12916-022-02720-7 (PMC9851584; doi:10.1186/s12916-022-02720-7)
Supplement: Supplementary file 9 — Additional file 9: Results of ITS analysis - Effect of pandemic on primary care consultations by 18-category ethnic groups. Table S1. Results of ITS analysis - Effect of pandemic on total consultations, within the multimorbidity population – Heterogeneity within ethnic group. [file 12916_2022_2720_MOESM9_ESM.docx]

**Additional File 9 – Results of ITS analysis - Effect of pandemic on primary care consultations by 18-category ethnic groups**

The ITS model was re-run for each White, Black, Asian, Mixed and Other within the multimorbid population, this time interacting the variables of interest with the more comprehensive 18 ethnicity categories from the 2011 Census to test for heterogeneity within each of the five overarching ethnic groups (**Table S1**). Only the estimates for the variables of interest (pandemic parameters) have been reported.

**Table S1. Results of ITS analysis - Effect of pandemic on total consultations, within the multimorbidity population – Heterogeneity within ethnic group**

|  | **Change in level after pandemic** | **Change in trend after pandemic** | **Number of Observations, % of multimorbidity population** |
| --- | --- | --- | --- |
| *White (Baseline = British)* |  |  | 1,158,975, 36.6% |
| Irish | 0.942 | 1.002 | 77,155, 2.4% |
|  | (0.100) | (0.004) |  |
| Gypsy or Irish Traveller | 0.252  (0.265) | 1.049  (0.037) | 917, 0% |
| Other White | 0.883**  (0.043) | 1.004**  (0.002) | 497,554, 15.7% |
| *Black (Baseline = African)* |  |  | 406,873, 12.9% |
| Caribbean | 0.978  (0.058) | 1.002  (0.002) | 378,143, 12.0% |
| Other Black | 0.809**  (0.067) | 1.009***  (0.003) | 129,601, 4.1% |
| *Asian (Baseline = Indian)* |  |  | 56,421, 1.8% |
| Pakistani | 1.048 | 0.998 | 32,728, 1% |
|  | (0.182) | (0.006) |  |
| Bangladeshi | 0.983 | 0.999 | 24,218, 0.8% |
|  | (0.188) | (0.006) |  |
| Chinese | 0.631* | 1.009 | 25,131, 0.8% |
|  | (0.129) | (0.007) |  |
| Other Asian | 0.958 | 0.999 | 87,546, 2.8% |
|  | (0.132) | (0.005) |  |
| *Mixed (Baseline = White and Black Caribbean)* |  |  | 58,805, 1.9% |
| White and Black African | 1.311  (0.262) | 0.990  (0.007) | 28,532, 0.9% |
| White and Asian | 1.060  (0.288) | 0.997  (0.009) | 13,621, 0.4% |
| Other Mixed | 1.052  (0.173) | 0.996  (0.006) | 54,926, 1.7% |
| *Other (Baseline=Arab)* |  |  | 3,930, 0.1% |
| Any other ethnic group | 0.416*  (0.178) | 1.026  (0.015) | 78,206, 2.5% |

Standard errors in parenthesises. ***p-value <0.001, **p-value<0.01, *p-value<0.05, **∙** p-value<0.1. Estimates are expressed as IRRs relative the baseline. Models only included the multimorbid population.
